# Economic impact case study of a wearable medical device for the diagnosis of obstructive sleep apnoea

**DOI:** 10.1186/s12913-024-11694-6

**Published:** 2024-11-01

**Authors:** Jo Setters, Jonathan Paynter, Jo Hanlon

**Affiliations:** 1 York Health Economics Consortium, York, UK; 2 Imperial College London, London, UK

**Keywords:** Sleep apnoea, Economic evaluation, Home sleep apnoea test, Cost savings, Diagnostics

## Abstract

**Background:**

AcuPebble SA100 (‘AcuPebble’) is a novel wearable medical device to diagnose obstructive sleep apnoea (OSA). This paper investigates the potential economic impact of the technology in the UK through cost savings analysis, and the redirection of savings into further diagnoses.

**Methods:**

A cost comparison study was conducted, comparing AcuPebble to the standard diagnostic approach of home respiratory polygraphy (HRP) and in-clinic polysomnography (PSG), estimating the net benefit value (NBV) and return on investment (ROI). Cohort size was varied to model the effects of volume discounted pricing and staff training costs. To demonstrate the potential for cost savings, data on the healthcare costs of undiagnosed OSA patients were used to quantify the benefit of increased OSA diagnosis rates, as facilitated by AcuPebble.

**Results:**

For 500 uses of AcuPebble, the NBV in the diagnostic pathway over one year would be in excess of £101,169, increasing to £341,665 for 1,500 uses, £1,263,993 for 5,000 uses, and to £2,628,198 for 10,000 uses, with ROIs of 2.02, 3.03, 5.05, and 6.56, respectively. Given an initial cohort of 1,500 patients, 4,555 extra AcuPebble studies could be completed by redirecting resources from HRP/PSG. Direct cost savings to the NHS from resultant lower undiagnosed rates could be between £24,147 and £4,707,810, based on the cost per use and the percentage of tests that result in a positive diagnosis (varied from 25 to 75% positives).

**Conclusions:**

AcuPebble presents an opportunity for substantial healthcare savings, enabling an increase in the number of people tested, diagnosed and treated for OSA.

## Background

Obstructive Sleep Apnoea (OSA) is a highly prevalent condition, impacting more than 936 million adults aged 30–69 globally, with 425 million experiencing moderate to severe disease [1]. Those with OSA often suffer from excessive daytime sleepiness, posing risks to productivity and quality of life [2]. They also face a 3 to 6 times higher risk of motor vehicle accidents [3]. Additionally, OSA is linked to various health issues, including high blood pressure, ischemic heart disease, and dementia [4]. Left untreated, OSA raises the risk of all-cause and cardiovascular mortality, emphasising the importance of effective treatment [5]. Beyond the physical and emotional toll, OSA imposes a substantial financial burden, encompassing direct costs of diagnosis and treatment and significant indirect costs [2], estimated as 95% of the total $115B cost in the US [2], or as much as 99% (€32B indirect to €234 M direct) in Italy [6]. Unfortunately the vast majority of individuals suffering from OSA are not diagnosed [2], partly due to the fact that diagnosis methods are complex and capacity in sleep clinics is severely limited (e.g., 6-month or longer delays from referral to sleep study [7]).

However, the importance of diagnosing and addressing OSA in adults can no longer be understated [8]. In the UK, the National Institute for Health and Care Excellence (NICE) has recently published a guideline for obstructive sleep apnoea/hypopnoea syndrome (OSAHS), recommending that diagnosis is carried out with a home respiratory polygraphy (HRP) test [9], in which the clinic provides the monitor and trains the patient in its use. When an HRP test is impractical, or additional monitoring is needed, then in-clinic polysomnography (PSG) should be used. Whilst both methods are used to record one or two nights of data, the clinic provides a more controlled environment for testing. Setup and surveillance are handled by clinicians, enabling a more comprehensive array of information to be recorded overnight, such as a camera feed and an electroencephalogram. Though it remains the gold standard in terms of OSA diagnosis, especially in more complex cases, it is more resource intensive and there is evidence to suggest that HRP is cost saving and non-inferior in terms of patient outcomes (e.g. [10]).

As well as providing lifestyle advice relating to tobacco, alcohol and weight, the recommended initial treatment for people with moderate and severe OSA is the use of a continuous positive airway pressure (CPAP) machine. For moderate or severe cases where CPAP is not appropriate, other treatments include a mandibular advancement device, or surgery [9].

**Fig. 1:**
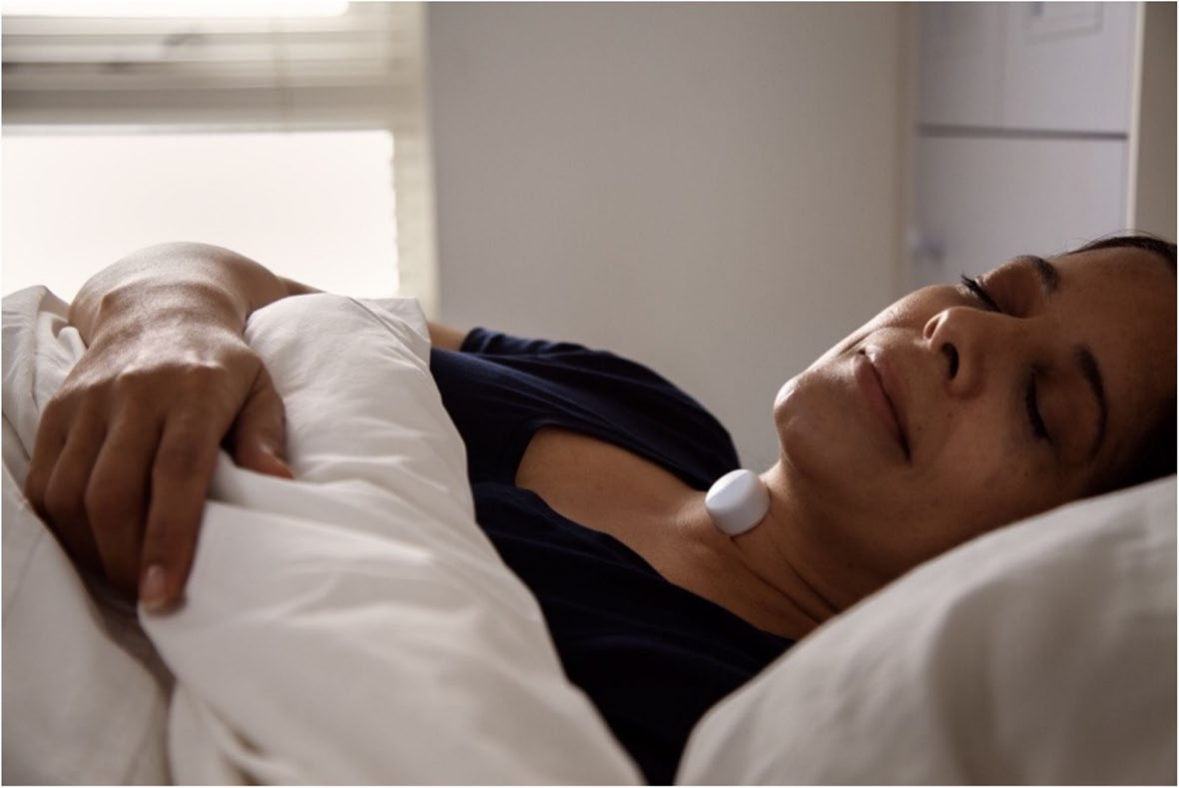
AcuPebble SA100 in use by a sleeping patient. Obtained with permission from https://acurable.com/en

AcuPebble SA100 (EU) (from here on referred to as ‘AcuPebble’) is a commercial innovative wearable device developed by Acurable for the diagnosis of OSA [11], in active use both widely within the UK public healthcare system (i.e. the National Health Service (NHS)), and internationally. The system has three main components: a neck sensor, a mobile application and a web application. The neck sensor and the mobile app are used by the patient to conduct the sleep study. The web application is used by healthcare professionals to design sleep studies and to review results. The neck sensor device is based on acoustic sensing technology which can detect internal body sounds produced by physiological processes such as respiratory and cardiac function; and, although AcuPebble can detect oxygen desaturation events with only the sensor at the neck, a compatible oximeter attached at the finger can be used to collect further oximetry information. The AcuPebble sensor is placed with a proprietary adhesive onto the base of the person’s neck, just below the throat, and is worn overnight. This sensor wirelessly transfers signals to the mobile app, from which data is uploaded to a secure cloud platform. These data are then interpreted by algorithms which use established parameters for the diagnosis of OSA. A report on the diagnostic result is instantaneously sent to the appropriate healthcare professionals. A photo of the sensor is shown in Fig. 1.

The neck sensor is powered by a battery that is charged between uses. The device can be used around 500 times with a single battery, until the battery loses capacity. The sensor is charged between uses with a standard micro-usb cable and the adhesive on the sensor also needs to be replaced. The battery charge and adhesive replacement can be done by a clinician, or by a patient if they have the sensor for a multi-night study. In a trial of AcuPebble based on 182 patients referred for OSA diagnosis in a UK hospital, it was shown to have sensitivity and specificity comparable to the standard approach to diagnosis (sensitivity, specificity, and prevalence with 95% CIs respectively: 92.73% (82.41, 97.98), 96.84% (91.05, 99.34), 36.67% (28.96, 44.92)). The vast majority of patients in the trial also reported that they found it more comfortable and easier to use than an HRP device [11]. A more recent study of 60 consecutively recruited patients and conducted in a Spanish hospital evaluated AcuPebble against manually scored PSG, resulting in a sensitivity and specificity of 92.86% (76.50, 99.12) and 97.14% (85.08, 99.93) respectively, with a prevalence of 44.44% (31.92, 57.51) [12].

In a recent systematic review of home sleep apnoea testing devices [13], level three devices (L3 - a category to which AcuPebble belongs – record a reduced set of signals from PSG and are conducted at the patient’s home) were overwhelmingly found to be cost effective and non-inferior to in-laboratory PSG. The evidence also suggests that waitlists are drastically shortened through the introduction of home testing (in one case [14] the average waiting time was 190 days shorter for patients receiving home testing over PSG). Whilst AcuPebble is indeed an L3 device, many established at-home testing devices (e.g., Apnealink Air, ResMed; Embletta MPR, Natus Medical) resemble miniaturised subsets of laboratory PSG equipment and employ similar signal acquisition sources and methods. Bespoke algorithms and acquisition techniques in devices such as AcuPebble may lead to distinct benefits and drawbacks, so the economic results of generic L3 evaluations may not translate exactly. As such, there are no existing directly applicable economic evaluations of AcuPebble. Furthermore, understanding that any savings made in each diagnosis may not be cash-releasing, this case study demonstrates the effect of extra tests due to reinvestment of savings as a reduction in healthcare demand.

This paper explores the potential economic impact of adopting a technology with the characteristics of AcuPebble within the UK public healthcare system (NHS). The case study reported in this paper uses a cost comparison approach, comparing AcuPebble to the standard diagnostic approach of HRP and in-clinic polysomnography. The cost per use of AcuPebble varies according to the level of use, so an additional analysis is presented of the net impact of four use and cost levels. A *de novo* analysis of the full implications of increased numbers of diagnosed cases on total treatment costs and benefits, is beyond the scope of this work. However, some economic consequences are considered following the main analysis here, based on a previously published economic model [15].

## Methods

The economic analysis for this case study considers the net incremental cost impact on the diagnosis for OSA. AcuPebble costs and savings - relative to the current standard for the diagnosis of OSA - were analysed in two distinct approaches. First, the difference in cost between an equivalent number of tests with AcuPebble and the current standard was estimated. Second, by redirecting these savings into further tests, the benefit could be quantified through the reduced lifetime cost of every additional OSA positive patient diagnosed and treated. Hence, this analysis assumes a lifetime horizon and a cost perspective of the NHS and personal social services. Costs were evaluated for a range of test uses, as the service model offered by Acurable discounts higher testing rates.

### Formulae

Net Benefit Value (NBV) is the total benefit of an investment or decision after accounting for its costs. Return on Investment (ROI) is a metric that represents the NBV as a proportion of the total cost.

$$\begin{array}{c}\:\begin{array}{c}\text{NBV}=\:\left(\Sigma\:\:\text{Total}\:\text{discounted}\:\text{benefits}-\Sigma\:\:\text{Total}\:\text{discounted}\:\text{costs}\right)\\\text{ROI}=\:\left(\Sigma\:\:\text{Total}\:\text{discounted}\:\text{benefits}-\Sigma\:\:\text{Total}\:\text{discounted}\:\text{costs}\right)/\:\Sigma\:\:\text{Total}\:\text{discounted}\:\text{costs}\\\:\:\:\end{array}\end{array}$$

In the context of this study, the ROI is used to represent the value of using AcuPebble to a health service (i.e., reduction in per-test cost) as a proportion of the costs (per-test cost of AcuPebble). This formulation assumes that patients’ health outcomes after diagnosis with AcuPebble are equivalent to the current standard, though presently there is only evidence on its diagnostic accuracy.

### Intervention costs

#### Fixed testing volumes

There are two principal pricing modalities for AcuPebble: a service licence and a product licence model. Under the service licence, the diagnostic process is managed by Acurable. They send the device to patients, receive and analyse the data and send results to referring doctors. This is the model most often used in the UK. Under the product licence, the NHS Trust receives the devices from Acurable and they manage the whole diagnostic process themselves. The manufacturers disclosed that the cost charged for this service varies according to a number of factors, including the cost of competitors in any given market. They did not confirm the exact pricing, but they did confirm the assumption that the cost for an NHS Trust under the service licence would be a maximum of £75 per use for 1500 uses.

We assume that an NHS Trust using this system would make a choice of their preferred licence type based on their capacity for managing the process and the logistics of using AcuPebble. In light of this, and the fact that we do not have information on a trust’s costs for managing the process internally, this case study has adopted a conservative approach by considering only the service licence approach in the calculations. When a new site begins to use AcuPebble, staff training on the system is provided by videoconference or through videos. The manufacturers reported that training takes about 30 min with a focus on demonstrating the different functionalities offered by the system to whomever is going to use it. They indicated that training might typically be attended by one consultant and one or two physiologists/nurses, for a sleep unit with 1,500 patients per year. They also reported that 2–3% of tests with AcuPebble may have to be repeated. Tests needing to be repeated are generally due to the sensor detaching from the patient’s skin during the night. In such a case, the mobile app detects the issue and instructs the patient to repeat the test. As a result, it should not require the patient to return to the clinic or need any additional clinician time.

The costs for one year of use of AcuPebble, based on these assumptions, are presented in Table 1. These resource costs are based on the assumption that a repeat test will have no additional cost, as the patient does not need to return to the clinic, nor do clinical staff need to carry out any additional actions. However, if a return to the clinic were necessary and a repeat test had the same cost as an initial test, with 2.5% of tests being repeated, the total number of tests in one year would be 1,537, with a cost of £115,275 for the testing and £115,392 including training.

**Table 1:** Costs for one year of AcuPebble use, with a cohort of 1,500 patients, under the service licence

Metric	Description	Value
Training for consultant	1 consultant for 0.5 h at £123/hr^a^	£63
Training for physiologists	2 physiologists for 0.5 h at £52/hr^b^	£54
Cost per test	£75 per test for 1,500 patients	£112,500
Total cost for the year	Cost per test × number of patients + training	£112,617

^a^PSSRU. Unit Costs of Health and Social Care 2021. Consultant: Medical

^b^PSSRU. Unit Costs of Health and Social Care 2021. Hospital-based scientific and technical staff. Band 6; Prices in ‘value’ column adjusted to 2022 using NHSCII [16]

#### Volume based discounting

The manufacturers indicated that the cost of a test under the service licence model would vary according to the level of use. A cohort of 1,500 patients a year is used as the basis for the initial economic analysis, which the manufacturers have suggested would be a typical number of cases seen in a hospital-based sleep clinic in the UK. However, to account for variation due to locality and clinic scale, a range of cohort sizes have been presented. According to NHS England’s monthly diagnostics data [17], there were 205,331 total sleep studies for the 2023–2024 year. Averaged across the 64 listed NHS sleep clinics in England [18] there would be 3208 sleep tests a year (or 62 a week). Viewing May 2024 diagnostics and waitlist data for sleep tests grouped by healthcare provider, there are as many as 990 tests in a month, or lower and upper quartiles of 66 and 226 tests per month. Therefore, a range of values from 500 to 10,000 were modelled, the chosen cost for each level is as shown in Table 2.

**Table 2:** Cost per use of AcuPebble for different usage levels

Number of uses	Cost per use	Total costs, without training
**500**	£100	£50,000
**1**,**500**	£75	£112,500
**5**,**000**	£50	£250,000
**10**,**000**	£40	£400,000

Staff training costs will vary too; we make the assumption that the level of training indicated in Table 1 is a minimum level to start using AcuPebble, but that levels of use greater than 1,500 tests would require additional staff to be trained: triple that amount for 5,000 tests and double again for 10,000 tests.

### Comparator costs

As stated in the Background section, the standard diagnostic approach is at-home respiratory polygraphy (HRP) or in-hospital polysomnography (PSG). It is assumed (based on data obtained from a representative large sample of NHS units in the UK [19]) that around 90% of OSA tests are HRP and 10% PSG. Using most recent NHS National Schedule data (2021/2022), the corresponding unit costs to those used in the economic analysis prepared for the NICE Guideline [20] are:

- ▪ HRP: £212 per use (Healthcare Resource Group: DZ50Z; outpatient procedures unit cost).
- ▪ PSG: £787 per use (DZ50Z; elective inpatient unit cost).

It has been reported that around 15-20% of HRP tests need to be repeated, which is typically because the sensors detach during the night [21]. This may only be discovered once the results are received in the clinic and, therefore, would require the patient to return and undertake the whole process again, incurring a repeat test cost. In-hospital polysomnography does not normally need to be repeated, as the patient is attended by a nurse during the night and any detached sensors will be replaced. Therefore, the numbers of each type of test in a cohort of 1,500 patients are assumed to be:

$$\:\text{N}\text{u}\text{m}\text{b}\text{e}\text{r}\:\text{o}\text{f}\:\text{H}\text{R}\text{P}\:\text{t}\text{e}\text{s}\text{t}\text{s}=90\%\;\:\text{p}\text{a}\text{t}\text{i}\text{e}\text{n}\text{t}\:\text{c}\text{o}\text{h}\text{o}\text{r}\text{t}+17.5\%\;\:\text{r}\text{e}\text{t}\text{e}\text{s}\text{t}\text{e}\text{d}=\text{1,586}$$

$$\:\text{N}\text{u}\text{m}\text{b}\text{e}\text{r}\:\text{o}\text{f}\:\text{P}\text{S}\text{G}\:\text{t}\text{e}\text{s}\text{t}\text{s}=10\%\:\text{p}\text{a}\text{t}\text{i}\text{e}\text{n}\text{t}\:\text{c}\text{o}\text{h}\text{o}\text{r}\text{t}=150\:$$

Based on the above, the total costs of the assumed usual care approach to testing are presented in Table 3, for the same cohort of 1,500 patients over one year.

**Table 3:** Costs for one year of standard diagnostic tests, for a cohort of 1,500 patients

Metric	Description	Value
Costs of HRP tests	1,586 tests x unit cost of £212	£336,232
Costs of polysomnography tests	150 tests x unit cost of £787	£118,050
**Total cost for one year**		**£454**,**282**

### Reinvestment of savings

As OSA is widely underdiagnosed, there is the possibility that more people could be diagnosed with the condition, for the same amount of economic resources. This, in turn, creates the potential for higher total resource requirements when the costs of the full treatment pathway are considered for these additional cases. Calculating costs and benefits across the full treatment pathway is beyond the scope of this case study. However, an attempt has been made to calculate these costs for the treatment of OSA in the UK, based on published evidence [15]. This report considered the treatments that were recommended for OSA at that time (2014) and a range of costs related to the condition, along with the impact on health care use associated with OSA and the wider social impact.

These wider effects are not accounted for when only the costs of the tests themselves are considered. Any direct savings in diagnosis could instead be multiplied by reinvesting in further tests, each of which may lead to a newly diagnosed patient with associated treatment costs and benefits. Hence, as an alternative method to quantify cost savings, the sum value of these extra tests was estimated.

The treatment of OSA has the potential to reduce the incidence (and associated healthcare costs) of cardiovascular events, stroke and road traffic accidents. In addition, there are costs to society of untreated OSA, including the costs of reduced productivity due to absence from work and from early retirement of OSA patients, though these are not considered in this case study. The aforementioned report [15] estimated the direct costs to the NHS of treating OSA to be £822.48 per patient per year. The direct costs of not being treated, per OSA patient, were estimated to be £905.21 per year. This gives a reduction in direct costs per patient per year of £82.73 at 2012/13 prices. Adjusting for inflation using NHS cost inflation index (NHSCII; [16]) and hospital and community health service pay and price inflation index (HCHS; [22]) for years prior to 2015, this would be £95.54 as of 2022.

The number of additional tests that can be carried out can be calculated by taking the net benefit value for a certain number of tests and dividing it by the cost per test. This assumes that the resource saving from lower cost diagnostic tests is retained within the pathway and used to increase test numbers among undiagnosed people.

The reduction in direct costs per patient only applies to those with true positive results, and so savings will vary with the proportion of tests that result in true positives. Pre-test probability varies between populations and referral strategies, and a range can be observed in cohort prevalences in England (e.g. 48% [7], 62% [23], 36.67% [11]). HRP and PSG patient outcomes are assumed equivalent, and false negatives are assumed to impart no further cost. As such, a range of proportions were modelled as true positives, and - assuming that these patients adhere to their treatment - the total annual value is then calculated by multiplying the number of true positive tests by the reduction in direct costs.

## Results

### Baseline net benefit value and ROI

Using the calculations from the previous sections, and where the standard testing approach is replaced with AcuPebble (assuming patient outcomes are equivalent) the net result would be a benefit value of £341,665 in the first year, for an NHS trust using the service licence (benefits of £454,282 minus costs of £112,617). Similarly, for one year, for a trust using the service licence, the ROI would be:

$$\:\left(\pounds\:\text{454,282}-\pounds\:\text{112,6}17\right)/\pounds\:\text{112,617}=\text{R}\text{O}\text{I}\:3.03$$

The costs and ROI can then be recalculated for a range of test volumes, including also additional staff training costs where appropriate and discounting the cost per test as in Table 2. The net value of benefits and ROI are as shown in Table 4.

**Table 4:** Net benefit value and ROI of AcuPebble for different usage levels

Number of uses	Total cost of AcuPebble (Tests and training)	Total cost of PSG and HRP	Net benefit value	ROI
**500**	£50,117	£151,286	£101,169	2.02
**1**,**500**	£112,617	£454,282	£341,665	3.03
**5**,**000**	£250,351	£1,514,344	£1,263,993	5.05
**10**,**000**	£400,702	£3,028,900	£2,628,198	6.56

### Impact of increased testing due to reinvestment

The calculations above indicate that, in the UK, AcuPebble could enable diagnosis of OSA with lower resource input than the standard of care approach. These savings can be reinvested into increased levels of testing. With an estimated annual saving due to treatment of £95.34, the total annual saving would then vary according to the percentage of these tests that produced correct positive results, where those patients go on to receive and adhere to effective treatment. Table 5 shows the total saving under three different scenarios, for each usage level and cost per use.

**Table 5:** Total potential annual direct savings to the NHS from treatment of additional cases of OSA, based on different percentages of positive test results, for each level of usage and cost per use

Number of uses	Percent of positive tests	Number of additional cases identified	Total potential direct savings to the NHS
**500**	25%	253	£24,147
	50%	506	£48,293
	75%	758	£72,440
**1**,**500**	25%	1,139	£108,791
	50%	2,278	£217,583
	75%	3,416	£326,374
**5**,**000**	25%	6,320	£603,762
	50%	12,640	£1,207,524
	75%	18,959	£1,811,286
**10**,**000**	25%	16,426	£1,569,270
	50%	32,852	£3,138,540
	75%	49,278	£4,707,810

### Impact of varying cost per use

The calculations presented in this case study indicate that, for a cohort of 500 patients requiring diagnostic tests, its use could produce a net benefit value (without reinvestment) of £101,169 in one year. This is assuming a price of £100 per test. However, since there is the potential for the cost per use of AcuPebble to be lower than in the case study calculation, this could yield increased savings. For example, if the price per test were to be scaled by a factor of $\:X$, the saving at 500 uses would be the original NBV plus 500 of the extra unitised savings: $\:NB{V}_{500}=\text{\pounds101,169}+\text{\pounds}100\times500\times(1-\text{X})$. ROIs from direct savings have been recalculated accordingly for two other relative price points (0.75 and 0.5 the originally stated cost) and are shown in Table 6.

**Table 6:** ROI for each usage band and cost scale. X = 0.5 represents a halving in cost

Number of uses	ROI		
	*X*=1	*X*=0.75	*X*=0.5
500	2.02	3.02	5.02
1500	3.03	4.38	7.06
5000	5.05	7.06	11.08
10,000	6.56	9.07	14.09

## Discussion

Assuming equivalent outcomes for patients diagnosed with AcuPebble and with HRP/PSG, testing a cohort of 1500 patients with AcuPebble could release savings of £341,665, for an ROI of 3.03. As the staff costs are relatively negligible compared to the cost of the test itself, the observed increased change ROI is almost entirely driven by the volume-based discounting model. While the benefits of AcuPebble demonstrated in this case study may not produce cash releasing savings (depending on the organisation of the healthcare system), the released capacity may be used to carry out additional diagnostic tests. In this scenario, the additional cases of OSA identified could result in direct savings to the NHS across the treatment pathway of between £24,146 and £4,707,810, based on the cost per use and the percentage of tests that are positive, though these are potential savings that require patients to receive and adhere to their treatment, furthermore assuming that there is no difference in treatment allocation and adherence between patients diagnosed with either method.

In addition to these savings, the device could result in reduced time in hospital and reduced time off work for patients. This reduced time benefits patients, but also reduces the use of limited hospital resources, potentially enabling higher testing volumes and shorter waitlists. In a published study of the use of AcuPebble [11], 150 patients were provided with the device and smartphone app and all were able to use the system effectively with no in-person training. The same patients would be given 30 min of training to use the standard HRP diagnostic approach. The costs of staff training for an NHS trust starting to use AcuPebble are low and do not make a big impact on the results of these calculations. As a result, the net benefit will not change greatly from year one (including training costs) to subsequent years (when training may not be required). In addition, the costs of repeat tests with AcuPebble are assumed to incur no additional costs. Should there be additional costs for some of these tests then it will also make only a modest difference to these results, given an estimation of 2.5% repeat tests. This estimation may in fact be conservative, as the number of tests repeated due to a failure of the system in Devani et al. [11] was 0 (or 0.5%, if user errors - i.e. leaving the phone in another room - are considered).

In this analysis, outcomes were considered equivalent between HRP/PSG and AcuPebble so that only direct costs were compared. An analysis that additionally models the rates of false positives and negatives may find increased costs from unnecessary treatment and from untreated patients who continue to place an elevated burden on the healthcare system. However, OSA has high night-to-night variability (e.g., 49% patients change severity category between nights [24]), elevating the importance of evidence of long-term patient outcomes over point estimates of accuracy. Novel sleep tests such as AcuPebble may make multi-night tests (to reduce the effect of variability) more economically and practically feasible through reduced resource use; clinicians may also divert more complicated cases to laboratory testing at their discretion, or manually analyse the information channels produced by AcuPebble; usability and ease of distribution may increase accessibility of diagnosis to the broader population; laboratory testing can also significantly disrupt normal sleep patterns [25]. The net outcome of all these effects may be equivalent to outcomes with PSG and HRP. This is substantiated in part through evaluations of HRP against PSG: for example, in a trial of 307 patients in Spain, comparing the Apnealink Air L3 portable monitoring device against in-laboratory testing [26], the home-testing arm was found to have favourable cost-effectiveness, with similar health related quality of life, CPAP adherence, and incidence of cardiovascular events. These outcomes were observed despite the Apnealink Air’s sensitivity and specificity of 91% and 95% when measured against PSG – an accuracy level that AcuPebble exceeds in evaluation [12].

It is not possible, within the limits of this case study, to assess the robustness of the assumptions and calculations used in the published economic model of impact on the full OSA treatment pathway. However, the model estimates a relatively modest saving in direct costs for the NHS from treating, as opposed to not treating, a person with OSA. This implies that greater diagnostic capacity resulting from the use of AcuPebble would result in a reduction in total direct costs for the NHS, from increased numbers of diagnosed cases.

Whilst no prior analysis exists on the cost effectiveness of AcuPebble for sleep apnoea diagnosis, the conclusions reached in this case study mirror that of the broader research around home sleep apnoea testing’s cost effectiveness [13], in that it is at least associated with lower direct costs than laboratory PSG testing. In this case, savings were realised through the modelling of non-cash-releasing savings into further tests and lower downstream costs, though the total value of the savings are optimistic for reasons previously identified.

The broader significance of these results is in noting the impact of increased testing: economic analyses that model only unit costs do not speak to the savings multiplying effects of reinvestment, nor the practical limitations behind long waitlists. Devices that enable increased testing through reduced costs, then make it feasible through reducing resource utilisation, could stand to have an outsised public health impact.

### Limitations of the study

This analysis was developed with data that was obtained, partly from the manufacturer, and partly from public sources. The limitations of the analysis are as follows:

- Only one of two possible pricing modalities for AcuPebble is used in the analysis, on the supposition that healthcare centres (generally NHS Trusts in the UK) will assess the total cost implications and choose the option which is most advantageous for themselves. To consider the effect of the alternative product licence, total fees charged by Acurable may decrease, though clinics would need to arrange distribution, collection, and management of test kits. This could incur shipping costs, administrative labour costs, or limit the clinics potential to meet testing volume demand. Whilst throughput was not addressed, a range of costs per test were considered in this analysis, which may aid in understanding the potential returns under a different licence.
- The analysis of the impact on the full OSA diagnostic and treatment pathway is based on a single, published economic model and we have not assessed the robustness of the assumptions and calculations used in that analysis.
- The case study only presents a cost comparison analysis, making the assumption that patient outcomes are similar to those where HRP is used, which is challenging to ascertain as the sensitivity and specificity of diagnostic tests varies with the severity of OSA [27].
- The analysis of benefit due to reinvestment assumes patients adhere to their treatment, which is often unrealistic [28].

### Recommendations

There is no evidence on the long-term outcomes of patients diagnosed with AcuPebble, nor for many similar home sleep apnoea testing devices - potentially due to their relatively recent introduction. For instance, given inter-night variability [24] in OSA diagnosis, direct comparison of diagnostic output may produce imperfect estimates of efficacy. Equally, a new diagnostic pathway may change patients’ relationships and adherence to treatment. Furthermore, given the large waitlists and growing awareness of OSA, research is needed to inform on the effect of alternative testing methods on waitlists, testing capability, and efficiency. Studies may want to consider modelling maximum feasible testing throughput, or the comparative effect of test kit distribution by mail rather than in-person.

## Conclusion

AcuPebble is a novel home sleep apnoea testing device that may enable significantly more diagnoses under equal economic constraints.

## Data Availability

The data that support the findings of this study are available from the following locations: ▪ Unit costs for consultants and physiologists, taken from the 2021 PSSRU Unit Costs of Health and Social Care, which can be found at https://www.pssru.ac.uk/wp-content/uploads/2021/12/unit-cost-of-health-and-social-care-staff-2020-21.xlsx. ▪ Unit costs for respiratory sleep studies, taken from the 2021/2022 NHS National Schedule, which can be found at https://www.england.nhs.uk/wp-content/uploads/2023/04/2_National_schedule_of_NHS_costs_FY21-22_v3.xlsx. ▪ The NHS cost inflation index (NHSCII) can be found in the Unit Costs of Health and Social Care 2023 manual, Table 12.1.1, page 98, DOI: 10.22024/UniKent/01.02.105685. ▪ The hospital and community health service (HCHS) pay and price inflation index can be found in the Unit Costs of Health and Social Care 2020, Sect. 15.3, page 163, DOI: 10.22024/UniKent/01.02.84818. ▪ The NHS England data on monthly diagnostic waiting times and activity for 2023-2024, in addition to years prior until 2008, are available at https://www.england.nhs.uk/statistics/statistical-work-areas/diagnostics-waiting-times-and-activity/monthly-diagnostics-waiting-times-and-activity/.
